# Nephrocalcinosis in a Child with Sotos Syndrome: A Case Report of Contiguous Gene Syndrome Encompassing *NSD1* and *SLC34A1* Genes

**DOI:** 10.3390/jcm14228200

**Published:** 2025-11-19

**Authors:** Agnieszka Bargenda-Lange, Anna Jakubowska, Anna Medyńska, Jan Bajtek, Robert Śmigiel, Katarzyna Kiliś-Pstrusińska

**Affiliations:** 1 Department and Clinic of Pediatric Nephrology, Wroclaw Medical University (UMW), 50-367 Wroclaw, Poland; 2 Department and Clinic of Pediatrics, Endocrinology, Diabetology and Metabolic Diseases, Wroclaw Medical University (UMW), 50-367 Wroclaw, Poland

**Keywords:** nephrocalcinosis, Sotos syndrome, contiguous gene syndrome, chronic kidney disease

## Abstract

**Background**: Nephrocalcinosis, characterized by the deposition of calcium salts within the renal parenchyma, is frequently identified incidentally in pediatric patients and may be associated with underlying genetic disorders. Sotos syndrome, a rare congenital overgrowth condition associated with neurodevelopment delay and congenital defects caused by mutations or deletions in the *NSD1* gene, has been sporadically linked to renal abnormalities, including nephrocalcinosis. **Clinical presentation**: We report a case of a male patient with Sotos syndrome and concurrent nephrocalcinosis, in whom genetic analysis revealed a microdeletion of chromosome 5q35 with a 2.2 Mb deletion encompassing both *NSD1* and *SLC34A1* genes. The *SLC34A1* gene encodes the NaPi-IIa sodium–phosphate cotransporter, essential for phosphate reabsorption in the renal proximal tubule. Haploinsufficiency of *SLC34A1* is implicated in dysregulated phosphate and calcium homeostasis, predisposing to hypercalciuria and nephrocalcinosis. Longitudinal follow-up demonstrated biochemical stability, resolution of nephrocalcinosis, and preserved renal function, supporting the hypothesis of an age-dependent attenuation in NaPi-IIa function. **Conclusions**: This case underscores the relevance of contiguous gene deletions in shaping complex clinical phenotypes and highlights the importance of early wide clinical screening in patients with Sotos syndrome to mitigate long-term renal complications.

## 1. Introduction

Nephrocalcinosis is defined as the deposition of calcium salts within the renal parenchyma and is often identified incidentally during ultrasound examinations or in the evaluation of other renal pathologies. In pediatric patients, genetic factors play a pivotal role [1], with nephrocalcinosis frequently associated with various inherited syndromes.

One such example is Sotos syndrome (OMIM 117550, ORPHA:821), a rare genetic neurodevelopmental disorder characterized by excessive physical growth, distinctive craniofacial features, and variable degrees of intellectual disability. Genitourinary abnormalities are observed in approximately 15% of affected individuals. The most commonly reported anomaly is vesicoureteral reflux; however, cases of renal agenesis, cryptorchidism, hypospadias, hydrocele, and phimosis have also been described. The coexistence of Sotos syndrome and nephrocalcinosis has been reported only rarely. Sotos syndrome is caused by a heterozygous pathogenic variant in the *NSD1* gene, which occurs de novo in over 95% of cases [2].

This report describes a retrospective clinical case of a patient with Sotos syndrome and concurrent nephrocalcinosis. Owing to the nature of the study, formal ethical approval was not required. The study was conducted in accordance with the Declaration of Helsinki. Written informed consent was obtained from the patient’s parents for the publication of the presented data, including the photograph that allows for patient identification. Clinical information was obtained from laboratory and imaging studies conducted during the initial diagnostic hospitalizations and subsequently during routine nephrology follow-up visits over a 10-year observation period. Genetic analysis revealed a microdeletion of chromosome 5q35 with a 2.2 Mb deletion encompassing not only the *NSD1* gene but also the adjacent *SLC34A1* gene, which encodes the NaPi-IIa sodium–phosphate cotransporter. Such contiguous gene alterations have been proposed as an explanation for a broader phenotypic spectrum.

## 2. Clinical Presentation

### 2.1. The Neonatal and Infancy Periods

**Figure 1:**
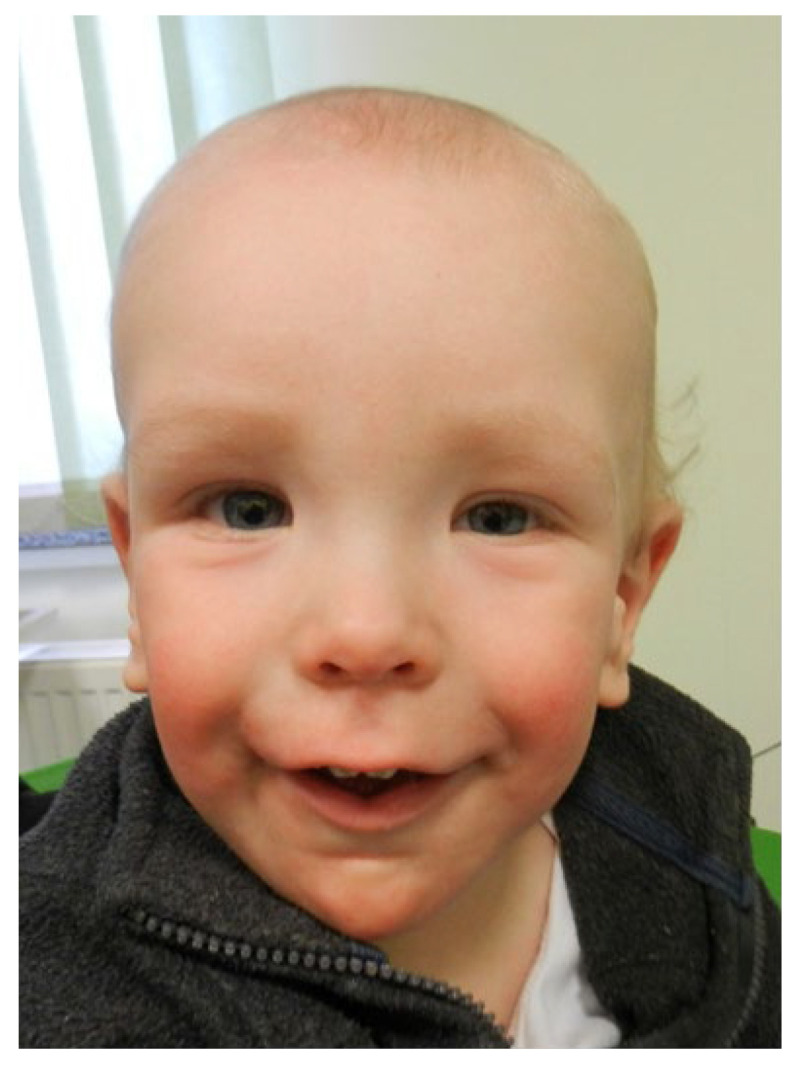
Typical facial features for Sotos syndrome in presented patient.

The boy was born following an uneventful, well-monitored pregnancy to non-consanguineous parents with no significant family history. Delivery was performed via cesarean section at 39 weeks of gestation due to suspected fetal macrosomia, with a birth weight of 3980 g (90th percentile according to Fenton growth charts). During the neonatal period, hypotonia and transient hypoglycemia were observed, along with dysmorphic features, including large hands and feet, coarse facial features, and macrocephaly (Figure 1). Ultrasound examination revealed nephrocalcinosis and mild bilateral dilation of the pyelocalyceal system. Vitamin D supplementation at a daily dose of 400 IU was initiated in accordance with current clinical guidelines for the general population. Based on the clinical presentation, a suspicion of Sotos syndrome was raised and subsequently confirmed by genetic analysis (MLPA P245), identifying a pathogenic variant in the *NSD1* gene (deletion of two markers for *NSD1* gene).

As part of a comprehensive multidisciplinary evaluation, the patient was diagnosed with a patent ductus arteriosus, corpus callosum hypoplasia, sacrococcygeal teratoma, and sensorineural hearing loss. During infancy, the patient exhibited psychomotor developmental delay.

### 2.2. First Nephrology Evaluation

At 2.5 years of age, the boy was referred to our department for further nephrological evaluation. Laboratory tests revealed normal serum levels of phosphate, calcium, parathormone (PTH), 25(OH) vitamin D, and 1,25(OH)_2_ vitamin D, as well as normal kidney function parameters. Hypercalciuria was diagnosed based on an elevated Ca/Cr ratio in relation to the age-adjusted reference range [3]. Phosphate handling parameters were within normal limits (Table 1 and Table 2).

### 2.3. Genetic Work-Up

The initial targeted NGS (next generation sequencing) panel included two genes implicated in idiopathic infantile hypercalcemia and phosphate–vitamin D metabolism: CYP24A1 and SLC34A1. The assay used was Illumina NextSeq500 (Star SEQ, Mainz, Germany), with paired-end 2 × 75 bp reads, which covered all coding exons with 10–20 bp of flanking intronic regions. The mean sequencing depth was 66× for CYP24A1 and 27.4× for SLC34A1, with a quality threshold >97–98%, as specified in the laboratory report. The initial molecular test showed no pathogenic SNVs/indels but indicated a possible heterozygous multi-exon deletion within SLC34A1. Therefore, a CGH array (comparative genomic hybridization) was undertaken. The confirmatory testing was carried out using the Agilent SurePrint G3 CGH ISCA microarray (Agilent, Santa Clara, CA, USA), a clinically validated oligonucleotide array designed for constitutional genomic testing. This platform contains approximately 60,000 probes distributed across the genome with enhanced coverage of regions associated with developmental disorders. In routine diagnostic use, the array provides an effective analytical resolution of approximately 300 kb for detecting copy number losses and gains, allowing reliable identification of pathogenic microdeletions within the 5q35 region. Using this platform, we identified and precisely delineated a heterozygous ~2.2 Mb deletion at 5q35.3 encompassing both NSD1 and SLC34A1.

### 2.4. Follow-Up and Longitudinal Evolution

Given the diagnosis and understanding of the pathomechanism of the disease, vitamin D3 supplementation was discontinued, and a normal-calcium, phosphate-rich diet was implemented. The patient has remained under regular nephrological follow-up and is currently 10 years of age. The patient is nonverbal and does not exhibit echolalia, but demonstrates preserved receptive language and is able to follow simple verbal commands. Examination reveals global hypotonia. He ambulates independently; however, significant valgus deformities of the feet and knees are present.

Throughout the observation period, the patient was maintained on a diet providing calcium and phosphorus intake appropriate for the respective age group, with efforts made to keep phosphorus intake within the upper normal range. Follow-up assessments revealed a decline in 25(OH) vitamin D levels, whereas other biochemical parameters—including serum calcium, phosphorus, PTH, and 1,25(OH)_2_ vitamin D—consistently remained within normal reference ranges. As episodes of hypercalciuria associated with dietary errors leading to excessive calcium intake were observed, cholecalciferol supplementation was introduced very cautiously, with close monitoring of biochemical parameters. No clinical signs of rickets were observed throughout the entire observational period.

Notably, since the age of eight, ultrasound examinations have no longer demonstrated the renal pyramidal calcifications that had been previously detected.

## 3. Discussion

Contiguous gene syndrome (CGS) refers to a genetic disorder resulting from the deletion or duplication of a chromosomal segment that includes two or more adjacent genes. Although these neighboring genes may have distinct biological functions, their simultaneous loss or gain can lead to a broad and variable spectrum of clinical manifestations, often affecting multiple organ systems with differing degrees of severity among individuals.

CGS is typically identified through advanced genetic testing techniques such as chromosomal microarray analysis or fluorescence in situ hybridization (FISH), which allow for the detection of submicroscopic chromosomal abnormalities, including deletions and duplications. Contiguous gene syndromes comprise genes located on autosomal or sex chromosomes. Autosomal contiguous gene syndromes (i.e., 22q11.2 deletion syndrome and WAGR syndrome) usually have characteristic clinical presentation and are not related to a specific metabolic profile [4,5]. Complex glycerol kinase deficiency is a contiguous gene syndrome caused by partial deletion of the Xp21 chromosome, which contains genes responsible for deficiency of the enzyme glycerol kinase (GKD, OMIM 307030), Duchenne muscular dystrophy (DMD, OMIM 310200), congenital adrenal hypoplasia (AHC, OMIM 300200), and intellectual disability (deletion in *IL1RAPL1* gene) [6,7,8]. Symptoms depend on the size of the deletion [7,9].

In the field of nephrology, CGS has been recognized as an underlying genetic mechanism in several well-characterized disorders. Examples include large deletions encompassing *TSC2* and *PKD1*, resulting in a combined phenotype of tuberous sclerosis complex and autosomal dominant polycystic kidney disease [10,11], as well as deletions involving *COL4A5* and *COL4A6*, which cause Alport syndrome with diffuse leiomyomatosis [12].

The co-occurrence of Sotos syndrome and nephrocalcinosis is rare. A limited number of reported cases suggest that microdeletions involving the 5q35 region—encompassing both the *NSD1* gene and the adjacent *SLC34A1* gene—could account for a broader and more complex clinical phenotype.

The SLC34A1 gene encodes the NaPi-IIa sodium–phosphate cotransporter, which plays a critical role in renal phosphate homeostasis by mediating the reabsorption of approximately 70–80% of filtered phosphate in the proximal tubules. Its dysfunction results in renal phosphate wasting and subsequent hypophosphatemia (OMIM 612286, ORPHA:244305). This condition suppresses the production of fibroblast growth factor 23 (FGF23), thereby relieving its inhibitory effect on the vitamin D-activating enzyme 1α-hydroxylase. The resulting increase in 1,25(OH)_2_ vitamin D levels enhances intestinal calcium absorption, promoting hypercalcemia and subsequent hypercalciuria, ultimately predisposing to the deposition of calcium salts within the urinary tract [13].

Renal involvement represents a central clinical feature in the spectrum of *SLC34A1*-related disorders, with severity largely determined by the type of mutation. Clinical features may include nephrocalcinosis, nephrolithiasis, tubulointerstitial nephritis, and progression to chronic kidney disease (CKD). Autosomal recessive mutations in *SLC34A1* are known to cause infantile hypercalcemia (IH) type 2 [14,15], a condition that typically presents in early infancy. By contrast, heterozygous mutations in *SLC34A1* have been associated with a milder phenotype, as suggested in previous reports [16].

Saugier-Veber et al. analyzed the genetic data of 116 patients with Sotos syndrome and observed that nephrocalcinosis occurred exclusively in individuals carrying microdeletion-type mutations [17]. Subsequently, Kenny et al. proposed a potential explanation by reporting two cases of children diagnosed with both Sotos syndrome and nephrocalcinosis, in whom genetic analysis identified a heterozygous deletion of the 5q35 region encompassing the *NSD1* and *SLC34A1* genes [18]. Similar findings were later described by Gonzales-Rodriguez et al., who reported a patient with a microdeletion additionally involving the *FGFR4* gene [19]. The genetic analysis of our patient aligns with these previously reported data. The abovementioned authors highlight an age-related attenuation in renal phosphate wasting, potentially reflecting a diminishing role of NaPi-IIa in phosphate homeostasis over time. Consequently, haploinsufficiency may present clinically predominantly during infancy—a critical developmental period characterized by elevated plasma phosphate levels and increased skeletal phosphate demands—when renal phosphate reabsorption is under maximal physiological strain [18].

In our patient, features of nephrocalcinosis were identified during infancy; however, a comprehensive evaluation of calcium–phosphate metabolism was not performed at that time. At the diagnostic assessment at 2.5 years of age, mild hypercalciuria was observed alongside otherwise normal calcium–phosphate metabolic parameters, which may support the proposed developmental changes in calcium–phosphate balance described above.

Given the pathophysiological background, we believe it is important to assess calcium–phosphate metabolism during infancy. In this context, we suggest screening for calciuria and phosphaturia by Ca/Cr and P/Cr ratio assessment every 3 months during the first two years of life, along with ultrasound evaluations every 6 months to monitor the presence of nephrocalcinosis in all children diagnosed with Sotos syndrome. If abnormalities are detected, we suggest a comprehensive evaluation of calcium–phosphate metabolism, along with expanded genetic testing for SLC34A1 mutations. In cases where an SLC34A1 mutation is identified, maintaining 25-OHD3 levels in the lower normal range appears advisable.

Janiec et al. analyzed long-term outcomes in patients with a history of infantile hypercalcemia, both with mutations in *CYP24A1* or *SLC34A1*, and demonstrated that these patients are at an increased risk of developing progressive chronic kidney disease. A reduced glomerular filtration rate (GFR) was noted not only in IH patients with recessive *CYP24A1* mutations but also in patients with a heterozygous *SLC34A1* variant, suggesting that elevated 1,25(OH)_2_D_3_ levels may contribute to kidney damage. However, individuals with heterozygous mutations typically showed a milder phenotype and better-preserved kidney function, indicating that additional factors influence CKD progression [20]. Similarly, Brunkhorst et al. demonstrated that monoallelic *SLC34A1* variant carriers exhibited stage 2 CKD in 50% of cases at the time of initial presentation and in 10.5% of cases at the final follow-up assessment [21].

Interestingly, the severity of nephrocalcinosis did not directly correlate with a decline in GFR. The authors suggest that progression to end-stage renal disease is more closely associated with tubulointerstitial inflammation and fibrosis than with mineral deposition alone. Although histopathological data are limited, the authors’ findings indicate the presence of chronic tubulointerstitial nephritis in affected individuals. The reason why this inflammatory response develops in some patients with IH remains unclear. Additionally, the data suggest that the severity of the initial kidney injury during an acute episode of IH plays a key role in determining long-term renal outcomes. Some patients also exhibited subtle, persistent disturbances in calcium metabolism, including elevated serum calcium levels and suppressed parathyroid hormone (PTH). This suggests that ongoing, subclinical disruptions in vitamin D metabolism may contribute to the development of microlithiasis and secondary tubulointerstitial inflammation [20].

Since the age of eight, no ultrasonographic evidence of nephrocalcinosis has been observed in our patient. Both serum creatinine levels and GFR remain within normal limits. However, transient episodes of hypercalciuria associated with dietary calcium intake have been noted. Given these findings, the patient needs ongoing clinical surveillance, including regular monitoring of renal function parameters, as well as the avoidance of additional risk factors that could contribute to the progression of chronic kidney disease.

## 4. Study Limitations

This study describes a single patient, which inherently limits the generalizability of the findings. As no kidney biopsy was obtained, a detailed morphological correlation with the clinical and imaging findings could not be established. Some early neonatal biochemical data are missing, which restricts the completeness of the initial metabolic and renal functional assessment. In addition, parental genetic testing was not performed.

## 5. Conclusions

To the best of our knowledge, only a few cases with contiguous gene syndrome encompassing NSD1 and SLC34A1 genes have been presented in the literature. Although the coexistence of Sotos syndrome and nephrocalcinosis is rare, it appears prudent to conduct a diagnostic evaluation of calcium–phosphate metabolism during infancy in all patients diagnosed with Sotos syndrome—particularly in light of the widespread recommendation for vitamin D_3_ supplementation. Early identification of metabolic disturbances may be essential for preventing the development of renal abnormalities and could potentially contribute to a more favorable long-term prognosis in the context of chronic kidney disease.

## Figures and Tables

**Table 1:** Blood parameters of the patient.

10.0	7.5	4.0	2.5	Age [years]
0.58 (0.52–0.69)	0.54 (0.52–0.69)	0.52 (0.44–0.65)	0.55 (0.39–0.55)	Creatinine [mg/dL]
145	139	118	98	GFR [mL/min/1.73]
0.85	ND	0.65	ND *	Cystatin C [mg/L]
4.2 (4.12–6.79)	4.7 (4.12–6.79)	4.8 (4.12–6.79)	5.8 (4.28–6.79)	Phosphate [mg/dL]
9.6 (9.16–10.52)	9.2 (9.16–10.52)	10.2 (9.16–10.52)	10.5 (9.16–10.52)	Calcium [mg/dL]
435 (141–460)	348 (156–369)	323 (156–369)	372 (156–369)	ALP [U/L]
36.1 (21.9–87.6)	50.2 (16.2–63)	37.6 (16.2–63)	50.3 (16.2–63)	PTH [pg/mL]
18.6 (17.36–48.4)	7.1 (17.36–48.4)	12.6 (17.36–48.4)	37.5 (17.36–48.4)	25(OH)D_3_ [ng/mL]
54.5 (42–95)	60.1 (42–95)	52.6 (42–95)	56.8 (43–140)	1,25(OH)_2_D_3_ [pg/mL]
23 (22–24)	23 (22–24)	25 (22–24)	23 (22–24)	Bicarbonate [mEq/L]

* ND—not determined. GFR, glomerular filtration rate (according to Schwartz formula); ALP, alkaline phosphatase; PTH, parathyroid hormone; 25(OH)D_3_, 25-hydroxy-vitamin D3; 1,25(OH)_2_D_3_, 1,25-dihydroxy-vitamin D3; (_) age-appropriate reference values [3].

**Table 2:** Urinalysis and ultrasound findings.

10	7.5	4	2.5	Age [years]
0.19 (<0.24)	0.15 (<0.25)	0.08 (<0.41)	0.74 (<0.5)	Ca/Cr [mg/mg]
1.2 (<1.37)	0.84 (<1.56)	0.9 (<1.75)	0.83 (<1.86)	P/Cr [mg/mg]
83	90	88	89	TRP [%]
1.13 (1.15–2.58)	1.37 (1.26–2.35)	1.36 (1.05–2.6)	1.67 (1.04–2.79)	TmP/GFR [mmol/L]
NC (−)	NC (−)	NC (+)	NC (+)	USG

Ca/Cr, urine calcium/creatinine; P/Cr, urine phosphate/creatinine; TRP, tubular reabsorption of phosphate; TmP/GFR, tubular maximum phosphate reabsorption/glomerular filtration rate; USG, ultrasound imaging; NC, nephrocalcinosis; (_) age-appropriate reference values [3].

## Data Availability

The analyzed datasets generated during the study are available from the corresponding author on reasonable request.
